# What Is the Value of a “Mountain Product” Claim? A Ranking Conjoint Experiment on Goat’s Milk Yoghurt

**DOI:** 10.3390/foods12102059

**Published:** 2023-05-19

**Authors:** Raffaele Zanchini, Giuseppe Di Vita, Luca Panzone, Filippo Brun

**Affiliations:** 1 Department of Agricultural, Forest and Food Sciences (DISAFA), University of Turin, Largo Braccini, 2, Grugliasco, 10095 Torino, Italy; raffaele.zanchini@unito.it (R.Z.); filippo.brun@unito.it (F.B.); 2 Department of Veterinary Sciences, University of Messina, Polo Universitario dell’Annunziata, 98168 Messina, Italy; 3 School of Natural and Environmental Sciences, Newcastle University, Newcastle upon Tyne NE1 7RU, Tyne and Wear, UK; luca.panzone@newcastle.ac.uk

**Keywords:** yoghurt, mountain, certification, claims, conjoint, willingness to pay, functional foods

## Abstract

Rural development is complex in marginal and disadvantaged areas, such as mountains, which impose high labour costs and restrict farmers in their choices of crop and livestock. To recognise this problem, the European Union regulates the use of the optional quality term “*Mountain product*” on the label. Consumers may recognise this label and be more willing to pay for it, resulting in higher revenues for producers using it. This study estimates the willingness to pay (WTP) for a mountain quality label. This WTP is then compared to that of functional and nutrition claims. For this purpose, we used a ranking conjoint experiment, using goat’s milk yoghurt—a typical mountain product—as a case study. Using a rank-ordered logit, we show that mountain quality labels generate a significant WTP, higher than that of functional claims. WTP differs by the demographic profile of the consumer. The study provided useful insights about the combination of the mountain quality label with different attributes. However, future studies are needed to adequately understand the potential of mountain certification as a supporting tool for farmers in marginal areas and for rural development.

## 1. Introduction

The literature has extensively investigated the role of quality signals, such as organic certification and geographical indications, as quality attributes capable of generating Willingness To Pay (WTP) and enhancing the value of food products [1,2]. However, few studies have focused on the novel optional quality term “mountain product”, which is related to the improvement of marginal areas condition [3]. This claim represents a strong opportunity for farmers working in marginal areas to differentiate and enhance their production [4]. In fact, in mountainous areas, farmers encounter greater difficulties than in the flatlands, such as high labour costs, difficulties in mechanisation, and limitations in cultivation and breeding choices [5,6]. Previous research indicates that label adoption is associated with an interest in the improvement of product value, product diversification, and consumers’ interest in healthiness and sustainable tourism in rural areas [7]. Finally, the literature also suggests that agriculture on mountains is unable to challenge conventional agriculture due to several environmental limitations, but it has the potential to attract consumers and enter niche markets, such as short food supply chains, earning higher market prices, and enhancing the value of local farmers’ products, becoming an expression of territorial development [3]. 

Based on these considerations, farmers in disadvantaged mountainous areas have been increasingly adopting this label, particularly in northern Italy, as shown in Table 1. Table 1 also shows that dairy products are an important category for the use of mountain claims, accounting for 31% of all products in Italy (38% in Northern Italy). Acceptance and interest in this label are increasing among farmers, with data from the Italian Ministry of Agriculture, Food Sovereignty, and Forestry showing an increase in the number of certified farmers from 615 in 2020 [8] to 1198 in 2023 [9].

The effectiveness of the mountain quality label as a tool to improve the conditions of farmers in marginal areas should also be evaluated from the perspective of consumers: the literature has not yet established if consumers are able to recognize the information carried by the label, as they might perceive the product as being of higher quality and be more willing to pay for it [10]. In particular, this novel attribute can support local development, providing mountain communities with benefits from direct sales of local products or sales in farmers’ shops, web shops, and farmers’ markets [11]. Previous research also showed this label is valued particularly by consumers who have a strong cultural identity and traditions associated with mountainous areas [12]. This study focuses specifically on the case of goat milk yoghurt, to determine whether the presence of a “mountain product” claim increases the value of this good. Based on European Regulations, processed food can adopt the mountain quality label, including yoghurt. Therefore, this certification can also be related to rurality and fits very well with yoghurt made from goat milk: in Italy, goat breeding is concentrated in mountain areas, due to the goat’s ability to make efficient use of resources in marginal areas [13]. This practice, as a result, has positive implications on the environment, protecting marginal areas, and traditions, which can evolve in the creation of slow food presidia for the protection of traditional foods [14].

Additionally, yoghurt can be classed as a functional food and can use health claims [15]. Compared with yoghurt from cow milk [16], which is more common, goat’s milk yoghurt is characterised by a higher amount of protein and dry matter. In addition, it contains 13% more calcium, 25% more vitamin B6, 47% more vitamin A, 134% more potassium, and, finally, 27% more selenium, an essential nutrient. The mountain quality label can be considered an environmental predictor [17], but it is also an important driver of food consumer behaviour, thus contributing to the success of a product in a market [18]. The role of mountain labels have been also investigated in combination with other food characteristics and labels [19]. This multi-labelling analytical approach can lead to interesting results since the presence of different quality attributes can modify consumers’ WTP [20,21]. Adopting mountain optional quality terms, in combination with other labels could help producers to deal with harsh market competition. However, the features to be presented should be chosen carefully, since combining different labels may lead to different results in terms of WTP [22,23].

One possible combination of attributes that can be used on a mountain yoghurt is the association with health claims. Within this context, understanding the relationship between the different EU quality labels becomes increasingly important. Moreover, the adoption of claims can increase farmers’ ability to differentiate and enhance the value of products [15]. The combination of the “mountain product” quality label with claims can also be justified from a technological point of view. Indeed, yoghurt can be considered a functional product, due to its intrinsic characteristics, such as the probiotic content [24]. Therefore, producers can adopt both claims to differentiate the product, gaining consumers’ attention in multiple market segments and obtaining a higher price for the processed product. Indeed, also for functional foods (functional foods), claims can be a strategic tool, since the communication of the properties and beneficial characteristics of these foods are regulated by the European Union [25,26]. According to European regulations, functional health claims communicate the physiological role of food constituents, while reduction risk disease claims inform about the capability of food to prevent or reduce certain health problems. In this work, we evaluated two claims in combination with the mountain quality label: a nutrition claim related to fibre content and a functional claim indicating the presence of probiotics in yoghurt. Such a combination represents a novelty, as the literature has not yet explored it. In addition, claims on fibre and probiotic content drive consumer attention to the positive role on human health of yoghurt consumption [27,28], which is also supported by regulations on the use of claims [25]. This study can detect whether the territorial link to mountainous and marginal areas can be as strong as the need of consumers to improve their health by buying foods. 

Based on this general consideration, the overall objective is to analyse the different perceptions of goat’s yoghurt attributes using a multi-attribute evaluation approach, represented by a ranking conjoint experiment [29]. Being the first attempt to measure WTP for a mountain claim, the aim of the study was to design a pilot study that could determine how consumers view and value this claim, rather than capturing a representative sample of the Italian population. For this purpose, a ranking conjoint experiment was carried out to evaluate the trade-off between the attributes and the utility conveyed by the mountain label and the two different health claims. Using a rank-ordered logistic regression, we were able to determine the WTP for the attributes of yoghurt, also linking this WTP to the socio-demographic characteristics of the consumer.

The remainder of this article is as follows: The following section presents the key literature on consumers’ preferences for mountain labelled foods and functional foods, including the research questions of the paper. Section 3 presents the ranking conjoint experiment and the econometric approach used in the analysis; Section 4 presents the main results, which are discussed in detail in Section 5. The last section concludes the study, highlighting key implications, limitations, and suggestions for future research.

## 2. Literature Review on Mountain Quality Label, Functional Foods and Research Questions

Yoghurt consumption provides several health benefits within a healthy diet [30]. ISTAT data indicates that the consumption of products derived from fermented milk in 2021 was 28.05 × 10^3^ t [31]. Different factors have been linked with yoghurt consumption: for instance, yoghurt consumption differs by gender and age [32], and geography [33]. The presence of an Organic label has been shown to increase the value of yoghurt, with a premium price ranging from 15 to 40% more than a conventional product, linked to beliefs over healthiness, environmental friendliness, quality, and safety [34]. Yoghurt is also considered by consumers as an environmentally friendly product [35]. In the next subsections, we will review the key literature on consumers’ preferences for mountain products, which will be linked and compared to that of functional foods.

### 2.1. Consumers’ Behaviour toward Mountain Labelled Products

The introduction of quality labelling on mountain products is recent and not yet established; indeed, very few studies have been conducted to assess consumer behaviour towards mountain-linked quality labels. Yet, the mountain quality label represents an important opportunity to support producers in marginal areas [3], and to recognise and protect mountains whilst ensuring consumers’ safety [7]. The possibility of certification is gaining interest among researchers and seems to be attractive to consumers [12,36]. The mountain label also seems to be strongly associated with the concept of *local* food products, and environmentally friendly food consumption [4], being perceived as more natural and characterised by marked cultural values and a strong territorial identity [37]. Indeed, consumers value mountain products for their ability to incorporate features and meanings that directly evoke the place where they are produced [38]. Research has shown that the label can contribute to the support of local economies in mountainous areas [12,39,40]. Indeed, a positive relation has been found between WTP for mountain milk with older consumers and environmental friendliness [41]. Moreover, Mazzocchi and Sali, (2022) [12] found that mountain products can be perceived as carrying positive values, generating a higher WTP than organic products, but lower WTP than some animal welfare claims, using a choice experiment. However, the interaction effect observed when such certificates are presented together with Protected Designation of Origin (PDO) certification is lower [19]. Based on this information, our first research question is:

**Research question 1** **(R1).** *Can the optional quality term “mountain product” be used to enhance the value of a goat’s milk yoghurt produced in marginal areas?*

The literature provides little information on the role of socio-demographic characteristics on preferences for mountain labels. For instance, a study on mountain labelled honey found that Italian consumers show a favourable attitude towards this label, with preferences varying across socio-demographic characteristics and lifestyles [4]. Recent research found that consumers associate this quality label with characteristics, such as safeness and tastiness, and respondents did not exclude buying mountain products [42]. Finally, Staffolani et al. (2022) [41] found that preferences for mountain quality labels are related to age and area of origin of consumers, but unrelated to education, income, or family size. Hence, our second result question is:

**Research question 2** **(R2).** *What is the role of sociodemographic characteristics, lifestyle, and health status, and consumer knowledge in predicting WTP for mountain quality labels and claims?*

It is worth pointing out that in Italy the use of a mountain logo to support rural mountain areas is regulated [43]. European regulations have provided a further certification that is generating great interest among academics, farmers and consumers, suggesting the great potential for rural development [5]. The European Commission has classified mountain certifications among the EU quality schemes, which include Geographical Indications and “other quality schemes” such as mountain labels and “Product of EU’s outermost regions”. The general objective of the UE with these certifications is to protect the names of certain products in order to promote their uniqueness, related to geographical origin, traditional knowledge, and links with disadvantaged natural areas [44].

The specific aim that prompted the European Union to regulate a quality label for mountain products was to provide a certification for producers in mountain areas that could be used to differentiate and enhance their products. Indeed, mountain areas are considered by the UE as marginal and disadvantaged, and the mountain quality label can support farmers in these areas [45]. Another important goal was to ensure consumer protection and improve living conditions in marginal areas [45]. According to Regulation (EU) No 1151/2012 [45], integrated with Regulation (EU) No 665/2014 [46], the optional *Mountain* quality term can be adopted by farmers when raw materials, animal feed and animal breeding are conducted in mountain areas. Regarding processed products originating from milk, such as yoghurt, certain accuracies should be taken to certify it as a mountain product [7]. Indeed, animals must be reared for at least two-thirds of their lives in the mountains, and the product must also be processed in the mountains. However, processing can be conducted outside when the distance from the place of origin does not exceed 30 km [46]. The procedure for adopting the quality term is regulated by the European Union [45,46]. Based on recent data, the implementation at the national level has been conducted by France, Germany, Italy, Romania, Slovenia, the Czech Republic, Bulgaria, and Croatia [8]. In Italy, farmers can adopt the mountain quality term after notifying local administrations, which add the firms to a regional database. The application and the implementation of European laws in Italy were led by the Italian Ministry of Agricultural, Food, Forestry, and Tourism Policy with the National Decree of 20 July 2018 [47].

### 2.2. Functional Foods and Functional Yoghurt

While a mountain claim is not commonly found on the labels of Italian yoghurt, products in the marketplace commonly make use of health and functional claims on food products. A product can be considered functional when it provides health benefits in a standard diet, through one or more constituents that have physiological effects [48,49]. Studies have been conducted on several differentiated food products, such as eggs [50], bread and cookies [22], fruit juices [51], milk [52,53], and dairy products [54]. Regarding the type of claim, Viscecchia et al. (2019) [54] suggested that function and risk reduction claims were both positively related by consumers; however, risk reduction claims produced a higher WTP. In addition, the difference between functional and nutritional claims was tested [55].

With regard to the drivers of functional foods consumption, many authors have highlighted the role of consumer beliefs, knowledge, and socio-demographic characteristics. In particular, beliefs about the properties and quality of functional foods are directly related to their consumption [15]. Moreover, authors have assessed the role of consumer knowledge with respect to functional foods consumption, showing that higher nutritional knowledge positively influences WTP and consumption [22,56]. Regarding the role of socio-demographic characteristics in the consumption of functional foods, a positive relationship was found with higher education [57], while the results are sometimes controversial with respect to age, with middle-aged consumers showing more interest in functional foods [58,59]. As for income, a positive interaction was found between higher income and functional food purchasing habits [60]. Finally, most of the literature has shown that women are the most interested and willing to pay more for functional foods and yoghurt [61,62].

Focusing on yoghurt as a functional food evidence related to consumers’ interest and behaviour toward this product were provided in the literature [63]. Due to its intrinsic characteristics, yoghurt can be considered a functional food in a standard diet and lends itself to the use of claims to communicate them to consumers [15,30]. Nutritional and functional claims concerning fat, sugar, fibre, vitamins, and calcium were evaluated differently, based on latent class analysis suggesting that most claims were positively evaluated [57]. Moreover, other studies have evaluated the role of probiotic claims, indicating that such information positively influences WTP for a functional yoghurt [62,64]. In terms of socio-demographics, nutritional and health claims are particularly interesting to middle-aged men [57]. 

Evaluating the information related to functional foods, the role of claims to communicate functional characteristics can be considered an important factor. The success of a functional product can also be affected by the communication of functional properties and constituents. Moreover, the combination of claims can lead to different results. Finally, consumers characteristics can be important as WTP predictors for both, functional characteristics and mountain quality labels, the following research question was considered:

**Research question 3** **(R3).** *Are there differences between nutrition claims, functional claims, and mountain claims in terms of WTP?*

## 3. Methods

### 3.1. Data Collection and Survey Design

Data collection was conducted using a multi-section questionnaire administered online. The questionnaire was constructed using Google Forms and shared by adopting a convenience sampling method, which can be considered a useful strategy to collect data exponentially, avoiding the limitations related to the distances among respondents within a population [65]. This method represents a non-discriminatory and non-probabilistic data collection method, which is also adopted in the case of topics that are difficult to investigate publicly [66]. In addition, it is better suited to the exploratory scope of the study, which aims to develop an initial understanding of an under-researched population.

Before disseminating the final version of the questionnaire, a pilot survey was conducted to evaluate the capability of respondents to understand the question and to deal with conjoint analysis. This preliminary step resulted in minor corrections of the sentences used to develop the questions [67].

The multi-section survey consisted of five parts based on general characteristics of yoghurt consumption; psychometric scales; intrinsic and extrinsic product characteristics; and, finally, socio-demographic and lifestyle characteristics and health status of respondents. Before starting the survey, participants had to give informed consent. Only consumers who agreed to be interviewed were allowed to proceed with the questions. Finally, the present study was approved by an academic ethics committee before being conducted. The data was collected in Italy at the end of 2022 and in the early months of 2023, resulting in a total of 289 valid questionnaires. While the number of observations may be small to generalise the results of this research, it is in line with previous literature using conjoint analysis [68,69,70,71]. A power analysis using the Conjointly website [72] indicates that for a population of 60,000,000 people, setting a 90% confidence level, a 5% margin of error, and a 0.5 sample proportion (all fairly standard criteria) leads to a recommended sample size of 271 respondents, an indication that a sample of 289 respondents has sufficient power for this study.

The summary statistics of the respondents are shown in Table 2.

Before starting the survey and after consent was given, a screening question was included to ask whether consumers would try goat’s milk yoghurt. Only consumers with an affirmative answer were directed to the conjoint analysis. 

The first part of the questionnaire was a conjoint experiment, which will be discussed below. The section on psychometric scales included the assessment of objective and subjective knowledge of yoghurt, an important predictor of consumer behaviour [73]. Objective knowledge represents an assessment of what consumers know about a certain topic or product, while subjective knowledge measures what consumers perceive they know [74]. To properly deal with the subject, the consumer knowledge assessment used by Pieniak et al. (2010) [75] was adapted to the current study.

### 3.2. Conjoint Experiment: Experimental Design

A multi-attribute approach was considered the most suitable method to evaluate and compare the role of mountain attributes for a niche product obtained in rural and marginal areas, with two health claims. Therefore, a ranking conjoint analysis was developed to assess consumers’ preferences and interests in various product attributes. The method involved a task in which consumers were asked to rank the conjoint cards generated by an orthogonal design from most favourable to least favourable.

To perform a reliable analysis, two aspects were considered and addressed: model efficiency and consumer response efficiency [76]. To improve the efficiency of the model and the reliability of the responses, an orthogonal design was generated. This design allows the number of cards to be reduced compared to the full factorial design, thus avoiding collinearity between attribute levels within the cards [77]. When the number of combinations is reduced, information can be easily conveyed, and consequently, respondents can handle the ranking task by improving the reliability of their answers [78].

Four attributes were used to generate the design, shown in Table 3, to maximise the efficiency of the analysis. The literature indicates that conjoint analysis should not exceed six attributes, and selecting fewer attributes can ensure better results [79]. Once the attributes were selected, they were combined to generate an experimental design, which generated eight hypothetical products with different characteristics.

The type of attributes was chosen to compare the role of the information provided by the optional *mountain* quality term and that conveyed by two claims: fibre content (nutrition claim) and probiotics (functional claim). The mountain attribute was evaluated using the optional quality label “mountain product” provided by the EU regulation No. 1151/2012. The claim related to probiotic content represents a functional claim, as referred to in EU Regulation No. 432/2012. Conversely, the fibre content claim was a nutritional claim, indicating only the presence of the functional component (EU Regulation 1924/2006). With regard to price, a market analysis was performed to identify three price levels. The average market price for 150 g of product was identified to determine the central value, while the others represent the standard deviation of the price distribution. Such combination of attributes represents a novelty aspect of this study, since, to the best of our knowledge, it is still unexplored [15,80].

**Figure 1:**
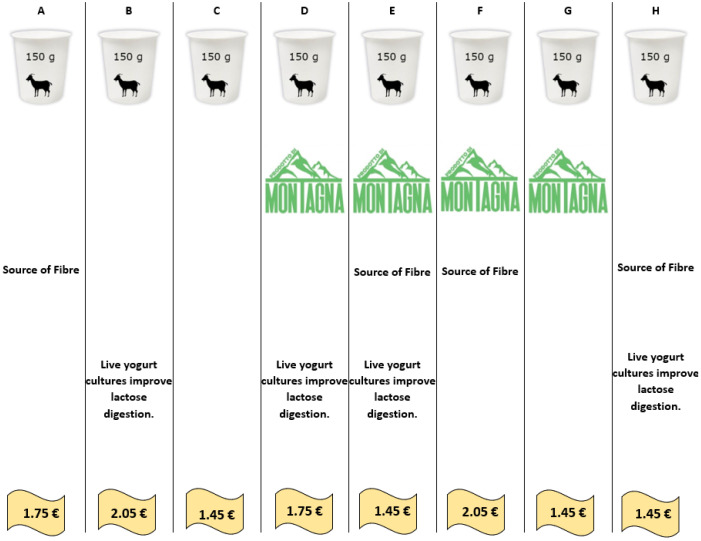
Example of experimental design as conjoint cards adopted in the survey. The columns are the different yoghurts, differing in their characteristics. Consumers had to rank the products from their favourite yoghurt to their least favourite one. The letters in this image are the reference codes used for the products.

The orthogonal design produced eight conjoint cards, depicted in Figure 1. Each card represents a yoghurt product. Consumers had the task to rank these eight products from most favourite combination to the least favourite. The method can be considered a stated preference method since the preference evaluation provided was hypothetical (i.e., consumers had no incentive to fully reveal their preference, such as having to purchase the top-ranked option). Once the cards were ranked by consumers, the data were elaborated using a Rank Ordered logistic regression to estimate WTP for the attributes [81]. This method was preferred to the OLS model since the latter has the main limitation of not being able to transform utility coefficients into a willingness to pay [29,82].

### 3.3. Econometric Analysis

The econometric model adopted is a rank-ordered logistic regression based on random utility theory [81] that can be adapted from a perspective of multi-attribute evaluation methods [83,84]. To this extent, the probability that one rank is followed by another can be obtained by considering a rank-ordered logit function, as illustrated in Equation (1). Consumer *j* perceives, from the combination of products, the utility ($U_{N\;}$) by evaluating several products *N* with *i* options, including different attributes, (price, mountain, fibre content, and probiotics), designated by the vector $Z_{i}$:

(1) $$p\left(U_{j1\;}\geq \;U_{j2\;}\geq \ldots \geq \;U_{jN\;}\right)=\overset{N-1}{\underset{i=1}{\prod }}[\;\frac{exp\left(Z_{i}\beta_{j}\right)}{\;\;\sum_{i}\;exp{\left(Z_{i}\beta_{j}\right)}_{\;}}\;]$$

where the utility function of the consumer equals:

(2) $$U_{ij}=Z_{i}\beta +\varepsilon_{ij}$$

with $\varepsilon_{ij}$ is the error term. Equation (2) can be rewritten to show the role of product characteristics in generating utility, as:

(3) $$U_{ij}=\beta_{1}P_{i}+\beta_{2}M_{i}+\beta_{3}F_{i}+\beta_{4}PR_{i}+\varepsilon_{ij}$$

where *P* = price; *M* = mountain; *F* = fibre; *PR* = Probiotics.

*WTP* can be obtained from Equation (3) as the marginal rate of substitution of a specific attribute and price. For instance, the *WTP* for the mountain quality term can be computed as:

(4) $$WTP=\;\frac{\partial P_{j}}{\partial M_{j}}=\frac{\partial U_{ij}/\partial M_{i}}{\partial U_{ij}/\partial P_{j}}=-\frac{\beta_{2}}{\beta_{1}}$$

To improve the model’s ability to describe *WTP* related to consumer characteristics, interaction terms were integrated into the previous equations.

The orthogonal design was conducted using IBM SPSS STATISTICS 28; the estimation was performed using the Stata 17.0.

## 4. Results

Table 4 shows the choice card presented to the consumers. The table also shows the predicted probability for each card being chosen as the preferred alternative during the task provided to consumers. At this stage, an important clue can be obtained about the ability of the combination of attributes to move consumers’ choices. The fifth option is the most favoured combination, with the product characterised by a low price, a mountain label and both nutritional and functional claims. In contrast, the combination with the lowest expected likelihood of being chosen as first is the third, with a low price and the absence of any differentiating attributes. These results suggest that product characteristics influence consumers’ choices.

Table 5 depicts the results of the ranking conjoint analysis, processed by means of rank-ordered logistic regressions. In detail, two regressions were run: *Model 1* describes the effects or the utility coefficients without the effect of consumers’ characteristics. *Model 2*, by introducing interaction variables, estimates the role of consumers characteristics on utility and WTP. The direction and effect are similar between the regressions. However, in Model 2, coefficients for food attributes are not significant, indicating that WTP for these attributes is consumers’ to consumers characteristics.

Concerning the price attribute, the negative coefficient indicates that as the price increases, consumers’ utility for goat’s milk yoghurt decreases. Price is the second most important attribute in terms of magnitude (in absolute value); indeed, only the mountain quality label obtained a higher coefficient. This outcome suggests that certain attributes may attract consumers’ attention and lead them to consider other characteristics rather than price alone. Another interesting indication provided by this analysis concerns the capability of mountain label to generate utility and, therefore, to attract consumers and as a differentiating tool for producers. Focusing on the functional component, both claims provide a positive utility to consumers. However, the functional claim concerning probiotic content was considered more important among the two, with a higher coefficient, thus providing the highest utility.

Model 2, in Table 5, shows the results of the ordered-rank logistic regression with the inclusion of interaction variables, which are useful for capturing the effects of covariates on the utility provided by the attributes of the conjoint experiment. Several variables related to socio-demographic, psychological, and lifestyle aspects were tested, allowing the detection of different drivers of consumers choices. Among socio-demographic variables, the only covariate showing an overall effect on yoghurt attributes is income: as income increases, the perceived utility of mountain, and fibre and probiotic claims also increase. Education only significantly influences the utility level of the probiotic content, decreasing the perceived utility of this attribute at high levels of consumers’ education. A possible explanation may be related to the fact that probiotics, as live yoghurt culture, may be naturally present in yoghurts, and educated people may perceive this attribute negatively because they expect the presence of live probiotics in yoghurt in any condition. Age covariate was significantly related to fibre content, indicating that older consumers are not interested in fibre-enriched yoghurt. The results also indicate that subjective knowledge has a negative relationship with the WTP for fibre in yoghurt, while no significant relationships were observed with mountain and probiotics. Objective knowledge was not associated with utility. Overall, the results suggest that knowledge is weakly related to yoghurt preferences. The analysis provides interesting results suggesting the role of lifestyle variables on consumers’ choices. The expected utility from fibre content increases as perceived health increases. Lactose intolerance is significant for fibre and probiotics contents only, as probiotic products have been seen to improve lactose digestion [46]. 

### WTP Estimates

**Figure 2:**
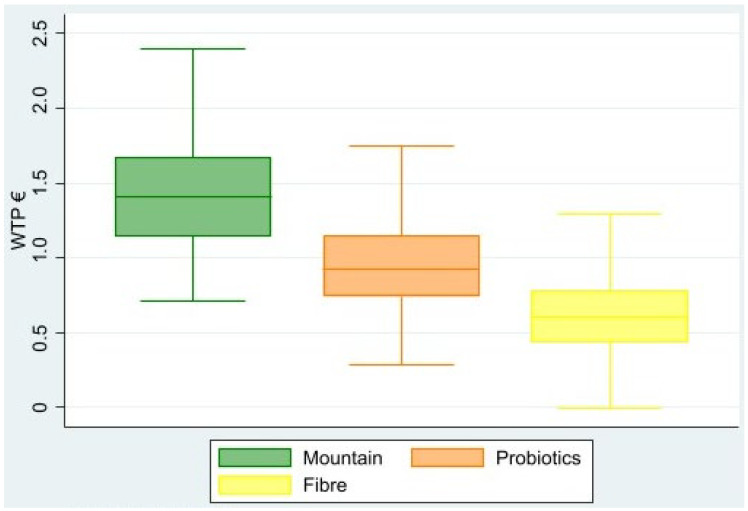
Estimated willingness to pay for the yoghurt attributes. The y axis represents the Willingness To Pay for the presence of an attribute. The distribution of WTP for food attributes is shown as boxplot, which show the interquartile ranges.

The coefficients obtained in the previous two regressions were used to estimate the WTP for the mountain claim (RQ1), and the functional claims (RQ3), as shown in Equation (4). Table 6 shows that the WTP obtained for all coefficients was significantly different from zero. Among the attributes, mountain certification is the most valued by consumers, generating a WTP of EUR 1.40 or EUR 1.42 when including consumer characteristics. This value is larger than that of other claims, with values of EUR 0.93–0.95 for probiotic content, and EUR 0.60–0.61 for fibre content. Figure 2 shows the estimated WTP value when consumer information was included (model 2), for each claim. This figure shows that the mountain claim is much larger than the other claims, and the fibre claim is the only attribute that obtained a WTP very close to 0 for some consumers. 

## 5. Discussion

This study determined the WTP for a mountain product claim on goat milk yoghurt, using a rank ordered logistics regression model, also estimating the WTP for fibre content and a probiotic claim. We found that the mountain quality label is a useful tool to enhance the value of yoghurt produced in mountain areas, and this quality label obtained the highest WTP. a positive WTP was found for fibre content and probiotic content, the latter receiving a higher WTP. 

Starting from the first research question (R1), the mountain label captured a significant WTP in goat milk yoghurt. This result is in line with other studies which found a high WTP for this attribute [19,85]. This is also in line with research on dairy products [86], as the mountain quality label is appreciated by cheese consumers and generates high WTP [19]. 

In line with the research question (R2), consumers characteristics predict WTP. Income represents the strongest predictor towards the quality signal evaluated in this paper, confirming previous studies that found a positive association among income, functional food, and functional claims used to communicate foods physiological effects [59,87]. The lack of link between income and a mountain label has not been found previously [4,41], and our result suggests that income is an important factor for this label, confirming the results of Bassi et al. (2021) [38], with wealthier consumers willing to pay more for this claim. The influence of age on WTP is in line with the current literature, suggesting that younger people are interested in functional characteristics [88] and negative relations were found significant toward health and nutrition claim on the label [89].

Education does not motivate the utility from mountain products, in line with current literature, in which this driver did not influence consumers behaviour towards the quality label [38,41]. Education represents a significant driver only for the probiotics content. The role of this diver is controversial in the literature on functional foods [15]. Indeed, several papers have found this characteristic not being significant for WTP for functional characteristics [62,88]. Education was found to be significant for lycopene content [90], but not for the functional component of milk communicated through nutritional claims [56]. Based on this literature, it is also possible that the role of education is strongly linked to specific products or characteristics and, therefore, difficult to generalise. Indeed, D’Antuono and Bignami (2012) [91] show that for typical food consumption, different aspects can be detected about education when is investigated in depth using the Food Neophobia Scale. In particular, education cannot have significant relationship with awareness while positive willingness to try traditional foods was detected.

Subjective knowledge—but not objective knowledge—was found to be significant and negatively related only with fibre content. The literature suggests that subjective knowledge can be a stronger predictor of consumers behaviour [92]. This result is not unexpected: the role of consumer knowledge as a driver of functional food consumption has been recognised in the literature [93]. However, when knowledge variables investigate specific characteristics of the products or functional components, it is easy to find significant relations [94]. In this paper, knowledge of yoghurt characteristics rather than functional characteristics was investigated, suggesting that general knowledge of a product can have different effects than knowledge of product-specific characteristics. 

Turning to lifestyle and health-related characteristics, a direct relation between health status and fibre content was found. This variable is often used to predict consumers behaviour towards functional foods and its role may differ depending on products and characteristics evaluated by researchers [15,95]. Concerning the relation with mountains, to the best of our knowledge, the role of health status in relation to the mountain quality label is still unexplored. Our results suggests that there is no relation or overlap between the mountain quality label and self-perceived health status. 

The current condition of having lactose digestion problems seems an important driver for WTP for different yoghurt attributes. Lactose intolerant consumers are more willing to pay for the fibre nutrition claim and for probiotic function claim. It is important that consumers can recognise the probiotic claim, as fermented milk products can be recommended to lactose intolerant consumers [96]. In a paper using yoghurt as a case study, a consumers segment indifferent to claims was found, mainly completed by respondents without health problems [57]. However, the literature also suggest that self-related health issues may also not be significant for WTP for nutrition and health claims [57]. An interesting consideration arises in this paper. Fibre content is valued more by intolerant consumers, which implies a certain misunderstanding of the role of fibre in yoghurt. Finally, health status is not significantly related with the mountain label, suggesting that health issues also do not generate an overlap between mountain certification and claims. 

Finally, the answer to the third research question (R3) shows that the nutrition fibre claims and the probiotic claims are valued less than the mountain claim. Moreover, the probiotic claim is considered more important than fibre content in goat milk yoghurt. This outcome is in line with the current literature on yoghurt, where fibre [57] and probiotics [64] were valued by consumers. The combination of these claims has not been found in the literature; however, similar results have been obtained, where in fermented milk products, probiotic claims are considered more important than the fibre claims adopted in the study [97]. Probably our consistent outcome may be due to the fact that probiotics are naturally contained in yoghurt [24] while fibre should be supplemented by generating a fortified product [98]. However, introducing fibre in yoghurt may attract particular consumers niches [57].

Indeed, the combination of quality attributes leads to different results than the presence of only one valuable characteristic [19,20]. The results suggest that the attributes are valued differently by consumers, with the mountain label being more important and in terms of utility and WTP, confirming what suggested in the literature about the potential of the mountain quality label to be appreciated by consumers [4,41]. Therefore, mountain quality labelling can be effectively adopted by producers in mountain areas and could lead to better WTP than the other claims tested here. In fact, information related to the marginality of products can reach consumers and be valued more than the healthy information conveyed by health claims. This result is in line with another study that found a mountain label to be more important than organic certification in PDO cheese [19]. However, other studies have compared the quality label with organic certification and animal welfare claims and found that other characteristics, in particular some animal welfare claims, can be appreciated even more, generating a higher WTP [12]. However, the validity of the term *mountain quality* is not discussed, since high WTP was also found for this attribute in different research [36,85]. In summary, it is possible to consider the mountain quality label as an effective tool to enhance the value of food products, but other attributes, not related to the marginality of food, can perform an even more important role. 

## 6. Conclusions and Implications

This is the first study exploring the role of the mountain optional quality label on goat’s milk yoghurt. It was found to perform an important role in enhancement of products developed in marginal areas, the optional quality term being able to produce a higher WTP than the other attributes. The results can support farmers’ decisions in adopting a tool provided by the Union to differentiate and enhance the value of marginal productions. The mountain quality label can be adopted in disadvantaged areas, resulting in higher revenues for farmers, which can be used to modernise a sector with important logistic and environmental challenges. Fibre and probiotics claims can also be adopted, but other investments would have to be made to produce yoghurt with these characteristics. Nevertheless, the mountain quality label alone seems to be the most effective way for producers to enhance the value of their products. Notably, our results suggest that the purpose of Regulations (EU) 1151/2012 and 665/2014 is relevant for the marketplace. 

Some limitations of the results obtained in this work should be noted. The main one is related to the sampling method. Indeed, convenient sampling provided a sample that has limits in generalising the results to the whole population. However, we believe that the novelty and the pilot nature of the work may mitigate the limitations arising from a convenient sample. Another aspect to be considered is related to the hypothetical bias. The experiment was hypothetical and therefore the results may overestimate the real importance imputable to the attributes investigated. Consumers may rank products differently in a real shopping scenario, where consumers have to pay for the products they prefer. In addition, another factor that can lead to an overestimation of the WTP is related to the sample that consisted of a market segment of interested consumers in goat milk yoghurt consumption. If we were to consider the entire market, including consumers not interested in goat milk yoghurt, WTP estimates would be lower. Future studies could be conducted in other European regions to confirm the results observed in Italy. Moreover, the same attributes could be evaluated in a cow’s milk yoghurt, to assess how the mountain quality label can be considered in a similar product. Finally, other relations between food attributes and consumers characteristics could be assessed. In particular, because the mountain quality label is strongly linked to territorial origin and tradition, future research could study the effect of the association with slow food presidia as a novel combination of attributes.

## Figures and Tables

**Table 1:** Number of certified products with “mountain product” claims in Italy.

	All Products	Dairy Products
South	202	36
Center	143	11
North	853	322
Italy	1198	369

Our elaborations are based on data provided by the Italian Ministry of Agriculture, Food Sovereignty, and Forestry [9].

**Table 2:** Summary statistics of the sample. *N* = 289.

Types	Variables	Categories	Per Cent	Mean (SD)
Lifestyle-health status	Lactose digestion problems	No	74.74	
		Yes	25.26	
	Self-evaluation of health status	1 to 7		3.70 (0.75)
Psychological	Objective knowledge	0 to 4		3.04 (0.79)
	Subjective knowledge	1 to 5		3.00 (1.09)
Socio-demographics	Age	18 to 78		41.19 (14.73)
	Education	Mid or lower education	6.57	
		High school	38.06	
		University	41.18	
		Higher education	14.19	
	Gender	Male	38.41	
		Female	61.59	
	Income	0 to 5500 EUR/month		2183 (1544)

**Table 3:** Attributes and attributes levels.

Attributes	Levels
Price	1.45 EUR/150 g; 1.75 EUR/150 g; 2.05 EUR/150 g
Mountain product	Yes; no
Fibre content	Yes; no
Probiotics	Yes; no

**Table 4:** Conjoint cards and predicted probability to be ranked as a most favourite product.

Cards	Price	Mountain	Fibre Content	Probiotics	Predicted Probabilities
1	1.75 EUR/150 g	No	Yes	No	0.027
2	2.05 EUR/150 g	No	No	Yes	0.028
3	1.45 EUR/150 g	No	No	No	0.019
4	1.75 EUR/150 g	Yes	No	Yes	0.176
5	1.45 EUR/150 g	Yes	Yes	Yes	0.472
6	2.05 EUR/150 g	Yes	Yes	No	0.088
7	1.45 EUR/150 g	Yes	No	No	0.088
8	1.45 EUR/150 g	No	Yes	Yes	0.102

**Table 5:** Results of the rank-ordered logistic regressions.

Variables	Coefficients Model 1	Standard Error	Coefficients Model 2	Standard Error
Price	−1.090 ***	0.135	−1.126 ***	0.138
Mountain	1.527 ***	0.096	0.709	0.957
Fibre	0.658 ***	0.063	0.334	0.552
Probiotics	1.023 ***	0.075	0.618	0.706
Mountain × Gender			−0.011	0.189
Fibre × Gender			0.034	0.125
Probiotics × Gender			0.087	0.152
Mountain × Income			0.203 ***	0.066
Fibre × Income			0.095 **	0.039
Probiotics × Income			0.148 ***	0.049
Mountain × Education			−0.188	0.133
Fibre × Education			−0.117	0.077
Probiotics × Education			−0.213 **	0.106
Mountain × Age			0.002	0.006
Fibre × Age			−0.007 *	0.004
Probiotics × Age			−0.001	0.005
Mountain × Subjective knowledge			−0.128	0.08
Fibre × Subjective knowledge			−0.107 *	0.059
Probiotics × Subjective knowledge			−0.001	0.059
Mountain × Objective knowledge			0.129	0.123
Fibre × Objective knowledge			0.054	0.082
Probiotics × Objective knowledge			0.108	0.096
Mountain × Health status			0.213	0.149
Fibre × Health status			0.219 **	0.092
Probiotics × Health status			0.064	0.115
Mountain × Lactose digestion problems			0.271	0.243
Fibre × Lactose digestion problems			0.302 **	0.148
Probiotics × Lactose digestion problems			0.454 **	0.180
Wald chi-square	283.07 ***		350.61 ***	

*, **, *** represent significant *p*-value at 0.1; 0.05 and 0.01, respectively.

**Table 6:** Willingness To Pay estimates.

Variables	WTP Model 1	Standard Error	WTP Model 2	Standard Error
Mountain	1.400 ***	0.172	1.417 ***	0.173
Probiotics	0.934 ***	0.121	0.948 ***	0.121
Fibre	0.604 ***	0.079	0.613 ***	0.080

*** represent significant *p*-value at 0.01.

## Data Availability

Data is contained within the article.
